# Adiposity and hyperglycaemia in pregnancy and related health outcomes in European ethnic minorities of Asian and African origin: a review

**DOI:** 10.3402/fnr.v57i0.18889

**Published:** 2013-02-28

**Authors:** Anne Karen Jenum, Christine Sommer, Line Sletner, Kjersti Mørkrid, Anne Bærug, Annhild Mosdøl

**Affiliations:** 1 Department of General Practice, Institute of Health and Society, University of Oslo, Oslo, Norway; 2 Department of Occupational Therapy and Orthotics, Faculty of Health Sciences, Oslo and Akershus University College of Applied Sciences, Oslo, Norway; 3 Department of Endocrinology, Morbid Obesity and Preventive Medicine, Oslo University Hospital, Oslo, Norway; 4 Institute of Clinical Medicine, University of Oslo, Oslo, Norway; 5 Department of Child and Adolescents Medicine, Akershus University Hospital, Lørenskog, Norway; 6 Norwegian Resource Centre for Women's Health, Oslo University Hospital, Oslo, Norway; 7 Norwegian Resource Centre for Breastfeeding, Oslo University Hospital, Oslo, Norway; 8 Department of Health, Nutrition and Management, Faculty of Health Sciences, Oslo and Akershus University College of Applied Sciences, Oslo, Norway

**Keywords:** obesity, gestational diabetes, pre-eclampsia, type 2 diabetes, cardiovascular disease, pregnancy, prenatal development, breastfeeding, ethnicity, immigrants

## Abstract

**Background:**

Ethnic minorities in Europe have high susceptibility to type 2 diabetes (T2DM) and, in some groups, also cardiovascular disease (CVD). Pregnancy can be considered a stress test that predicts future morbidity patterns in women and that affects future health of the child.

**Objective:**

To review ethnic differences in: 1) adiposity, hyperglycaemia, and pre-eclampsia during pregnancy; 2) future risk in the mother of obesity, T2DM and CVD; and 3) prenatal development and possible influences of maternal obesity, hyperglycaemia, and pre-eclampsia on offspring's future disease risk, as relevant for ethnic minorities in Europe of Asian and African origin.

**Design:**

Literature review.

**Results:**

Maternal health among ethnic minorities is still sparsely documented. Higher pre-pregnant body mass index (BMI) is found in women of African and Middle Eastern descent, and lower BMI in women from East and South Asia compared with women from the majority population. Within study populations, risk of gestational diabetes mellitus (GDM) is considerably higher in many minority groups, particularly South Asians, than in the majority population. This increased risk is apparent at lower BMI and younger ages. Women of African origin have higher risk of pre-eclampsia. A GDM pregnancy implies approximately seven-fold higher risk of T2DM than normal pregnancies, and both GDM and pre-eclampsia increase later risk of CVD. Asian neonates have lower birth weights, and mostly also African neonates. This may translate into increased risks of later obesity, T2DM, and CVD. Foetal overgrowth can promote the same conditions. Breastfeeding represents a possible strategy to reduce risk of T2DM in both the mother and the child.

**Conclusions:**

Ethnic minority women in Europe with Asian and African origin and their offspring seem to be at increased risk of T2DM and CVD, both currently and in the future. Pregnancy is an important window of opportunity for short and long-term disease prevention.

The rise in obesity prevalence, particularly childhood and early adulthood obesity, is estimated to substantially increase the costs for the health care systems in the EU (1). In 2010, between 35 and 80% of the adult population in European countries were overweight or obese (2). Together with sedentary behaviours, these conditions are the major drivers of the increased prevalence of type 2 diabetes mellitus (T2DM). Childhood obesity may also lead to a future epidemic of cardiovascular diseases (CVD) in early adulthood (3). These health problems are not evenly distributed, as most ethnic minority groups with origin from low- and middle-income countries, particularly from Asia and the Middle East, are disproportionally affected (4–6). Furthermore, T2DM is diagnosed up to 10–15 years earlier in the first generation of immigrants from Asia and the Middle East (including North Africa), compared with ethnic Europeans (5, 7), and mortality rates from CVD (8) and T2DM (9, 10) are higher. The pathophysiology of this increased risk is thought to be complex, involving a life history of gene and environment interactions, exacerbated by low physical activity and unfavourable diets (11). Migration may lead to a rapid ‘Westernization’ of dietary habits, typically an increase in intake of energy, fat, and refined carbohydrates and a shift favouring animal relative to vegetable food sources (12, 13). Some studies point to socioeconomic deprivation to a large extent explaining the ethnic variations in CVD risk (14–17), but to a lesser extent the high prevalence of T2DM (18). However, as many immigrant populations in Europe are rather young (19), the morbidity and mortality patterns of the ethnic minority groups are still emerging.

Pregnancy can be considered *a natural stress test* for the mother (20). Complications like gestational diabetes mellitus (GDM) and pre-eclampsia seem to be early markers of disturbances in glucose metabolism, endothelial dysfunction, and hypertension (21). In turn, presence of such factors predicts future risk of T2DM and CVD in the mother (20). A recent phenomenon is pregnancies complicated by pre-existing T2DM with numbers now exceeding that of type 1 diabetes in Europe (22). Also T2DM substantially increases the risks of several adverse pregnancy outcomes both for the mother and the child (23).

Research into the developmental origins of health and disease has established that a poor intrauterine environment may have significant consequences for both foetal growth and later risk of non-communicable diseases in the offspring (24, 25). Also foetal overgrowth, which for a long while has been observed with maternal type 1 diabetes (23), is associated with several adverse outcomes for mothers and offspring also in milder hyperglycaemia and obesity (26). Thus, the higher prevalence of T2DM in ethnic minority women of reproductive age, and possibly also variations in obesity prevalence, milder forms of hyperglycaemia, and pre-eclampsia, may have major health consequences across generations.

The aim of this article is to review the literature about differences between ethnic groups of Asian and African origin and the majority population in European countries, respectively, regarding the following topics: 1) adiposity, hyperglycaemia, and pre-eclampsia in pregnancy; 2) future risk for the mothers of obesity, T2DM, and CVD; and 3) prenatal development and possible influences of maternal obesity, hyperglycaemia, and pre-eclampsia on the child's future risk of obesity, T2DM, and CVD. Ethnicity may be defined as the social group a person belongs to because of a shared culture, history, geographical origin, language, diet, physical, genetic, and other factors (27). In this review, the focus is on populations of Asian and African origin, as they are widely represented (19). In some countries, this will also include the second and higher generations after immigration.

## Evidence acquisition

Literature was gathered through two different procedures. Systematic searches were performed in PubMed to identify European studies with a main aim to compare an outcome in ethnic groups of Asian and African descent compared with the general population/majority ethnic group. The selected outcomes were as follows:

1. adiposity/weight status in pregnant women (Table 1);
2. gestational weight gain (Table 2);
3. GDM (Table 3);
4. pre-eclampsia (Table 4);
5. post-partum weight retention (Table 2); and
6. birth weight (Table 5).

**Table 1:** Comparison of BMI in pregnant women between groups of Asian and African origin and the majority population in European countries

Author, year, (reference number)	Study design	Study population	Variable	Ethnic groups	n	Results		Comments
Djelantik et al. 2012 (39)	Community-based cohort study	Pregnant women living in Amsterdam, the Netherlands, January 2003–March 2004	Pre-pregnant BMI (pBMI)	N Dutch African Turkish Moroccan Other non-Western Other Western	7,871 4,900 495 328 585 904 659	pBMI 25–30 13.7% 30.9% 22.7% 33.4% 21.3% 11.1%	pBMI>30 4.1% 18.0% 11.0% 12.7% 7.9% 3.5%	Self-reported height and weight. pBMI>30 significantly more frequent among Turkish, Moroccan, and women of African descent
Jenum et al. 2012 (41)	Population-based cohort study	Healthy, pregnant women from three city district of Oslo, Norway, May 2008–2010	Pre-pregnant BMI (pBMI)	N Western Europe South Asia East Asia Middle East Somalia Sub-Saharan Africa/other non-Western	823^a^ 313 188 39 112 35 30	pBMI. Mean (SD) 24.6 (4.8) 23.7 (4.1) 22.3 (3.4) 25.9 (5.1) 26.8 (6.5) 26.3 (6.5)		Self-reported pre-pregnant weight, measured height at first visit. Heterogeneity between groups.
Hestlehurst et al. 2010 (37)	Database study	Women attending 37 maternity units in UK, 1989–2007	Early pregnant BMI	N White Asian/Asian British Black/Black British Mixed Chinese or other ethnic groups	619,323 447,423 50,738 22,525 5,962 11,394	BMI 25–29.9 24.7% 25.6% 32.3% 23.5% 20.5%	BMI>30 13.7% 10.4% 20.6% 12.7% 7.6%	Maternal height and weight at initial GP appointment, adjusted for gestational age. The study population was found to be nationally representative. Black/Black British had significantly higher ORs for overweight and obesity (Reference: White. Adjusted for age, parity, employment, and deprivation).
Ochsenbein-Kölble et al. 2007 (48)	Prospective cross-sectional study	Nulliparous women attending an obstetric prenatal outpatient clinic, Zürich, Switzerland, January 1996–February 2000	Pre-pregnant BMI (pBMI)	N Caucasians Asians Blacks	4,034 3,242 578 214	pBMI. Mean (SD) 23.6 (4.5) 22.5 (3.9) 24.1 (4.3)		Self-reported pre-pregnant weight. Mean pBMI sign different in Asians, but not in Blacks, compared to Caucasians.
Loetscher et al. 2007 (40)	Retrospective cohort study	Nulliparous women attending a prenatal outpatient clinic, Zürich, Switzerland, 1999–2003	Pre-pregnant BMI (pBMI)	N Switzerland Turkey Sri Lanka Middle East South Asia Far East Africa	1,432^a^ 262 95 173 24 36 36 118	pBMI>25 22.9% 42.1% 34.1% 41.7% 19.4% 2.8% 50.0%	pBMI>30 8.0% 16.8% 4.6% 8.3% 2.8% 0.0% 16.1%	Self-reported pre-pregnant weight, measured height at first visit. No analyses on differences in pre-pregnant BMI across ethnic groups were presented.
Hestlehurst et al. 2006 (38)	Database study	Women attending a maternity unit, Middlesbrough, UK, 1990–2004	Early pregnant BMI	N Caucasian Mixed race Asian/Asian British Black/Black British Chinese/other Ethnic group unknown	36,821 33,420 187 1,486 77 133 1,058	BMI 25–29.9 23.8% 18.2% 23.4% 28.6% 14.3% 24,1%	BMI>30 10.8% 11.2% 9.5% 10.4% 6.8% 11.2%	Maternal height and weight at initial GP appointment, adjusted for gestational age. Ethnicity was not significantly associated with obesity in models adjusted for age, parity, employment, deprivation, and marital status.

a Results from women of other European and American nationalities not reported in this table.

For adiposity/weight status in pregnant women, studies reporting this measure in baseline data were also included. Weight data from the general female population were excluded as few studies report data for women of reproductive age separately. The search terms are presented in Appendix 1. The search was limited to papers in English and within the last 10 years with the final search 1 October 2012. Relevant studies from non-European countries were included when European studies were sparse or the studies highlighted specific aspects of relevance to the paper's aims.

**Table 2:** Comparison of gestational weight gain and post-partum weight retention between groups of Asian and African origin and the majority population in European countries

Author, year, (reference number)	Study design	Study population	Variable	Groups by ethnicity	n	Results	Comments
Ochsenbein-Kölble et al. 2007 (48)	Prospective cross-sectional study	Nulliparous women attending an obstetric prenatal outpatient clinic, Zürich, Switzerland, January 1996–February 2000	BMI and weight gain centile curves during the pregnancy	N Caucasians Asians Blacks	4,034 3,242 578 214	Asians and Blacks had significantly lower weight gain than Caucasians over the whole pregnancy period (*p*<0.001).	Self-reported pre-pregnant weight. Weight measured at each antenatal visit. Absolute weight change not presented.
Loetscher et al. 2007 (40)	Retrospective cohort study	Nulliparous women attending a prenatal outpatient clinic, Zürich, Switzerland, 1999–2003	Gestational weight gain	N Switzerland Turkey Sri Lanka Middle East South Asia Far East Africa	1,432^a^ 262 95 173 24 36 36 118	Mean (SD) net weight gain 10.9 (6.0) 11.6 (7.5) 8.4 (5,2) 11.3 (5.3) 10.3 (4.6) 11.6 (5.2) 9.0 (7.1)	Self-reported pre-pregnant weight. Weight at delivery (within 1 week prior to delivery) measured at the hospital No analyses on differences in weight gain across ethnic groups were presented.
van Poppel et al. 2012 (82)	Prospective cohort study	Pregnant women attending antenatal care in Amsterdam, the Netherlands, January 2003–March 2004	Weight retention 3–5 months post-partum	N Dutch Surinamese Turkish Moroccan Ghanaian	4,212^a^ 2,601 231 171 172 42	Weight retention>5 kg 26% 27% 52% 33% 26%	Self-reported pre-pregnant weight and weight 3–5 months post-partum. Turkish women had significantly higher crude OR for weight retention>5 kg (Reference: Dutch)

a Results from women of other European and American nationalities not reported in this table.

For literature regarding mechanisms and theoretical models of relevance to highlight how the long-term health outcomes (part 2 and 3) may disproportionally affect ethnic minority groups of Asian and African origin, a hierarchical strategy was used. PubMed was searched using relevant search terms regarding a subtopic to identify: 1) review papers and theoretical reviews; 2) primary research papers of high quality (primarily cohort studies thought to be representative) if reviews did not provide specific information relevant for the ethnic minority populations in Europe; and 3) relevant reviews or primary research papers about ethnic differences from other continents, to be interpreted into a European context. Only the most recently published papers were used when relevant.

In the searches, papers on type 1 or type 2 diabetes and other specific diseases diagnosed prior to pregnancy, as well as papers about short time outcomes like stillbirth, caesarean section, malformations in the offspring, and about interventions, were excluded. However, as breastfeeding may benefit the mother and the baby in relation to several topics of interest in this review, this dimension of nutrition was included. As causal models rely on the available confounders and may make results from different papers less comparable, we primarily present crude or minimally adjusted results in the tables, to highlight the public health aspect. Although some risk factors for the clinical outcomes are mentioned, causal factors are not emphasised in this paper.

**Table 3:** Comparison of the risk of gestational diabetes mellitus (GDM) between groups of Asian and African origin and the majority population in European countries

Author, year, (reference number)	Study design	Study population	Variable	Ethnic groups	n	Results		Comments
Jenum et al. 2012 (41)	Population-based cohort study	Healthy, pregnant women, living in three city district of Oslo, Norway, 2008–2010	GDM with WHO criteria	N Western Europe (ref) South Asia Middle East Other minorities	759 313 188 112 146	Crude prevalence 13% 11% 15% 17% 12%	AOR *p*=0.026 – 2.24 (1.26–3.97) 2.13 (1.12–4.08) 1.45 (0.77–2.73)	AOR: adjusted OR, adjusted for age, pre-pregnant BMI, parity
			GDM with modified IADPSG criteria (no 1-h glucose values)	N Western Europe South Asia Middle East Other minorities	759 313 188 112 146	Crude prevalence 32% 24% 42% 37% 30%	AOR *p*<0.001 (ref) 2.94 (1.94–4.47) 1.79 (1.10–2.93) 1.44 (0.91–2.28)	AOR: adjusted OR, adjusted for age, pre-pregnant BMI, parity
Khalil et al. 2012 (62)	Cohort study	Pregnant women in 3 hospitals in South England, UK	Diagnosis with modified WHO criteria (fasting plasma glucose≥6.0 mmol/l)	N Caucasian Afro-Caribbean South Asian East Asian Mixed	76,158 57,564 11,395 3,645 1,793 1,761	1,355 cases (1.8%)^a^^b^	Crude OR (ref) 1.89 (1.66–2.16) 2.31 (1.90–2.80) 2.26 (1.72–2.96) 1.18 (0.81–1.70)	Data collected as part of routine antenatal care. GDM screening by 2-step approach
Makgoba et al. 2011 (63)	Retrospective study	Pregnant women in 15 maternity units in North West London, UK, 1988–2000	GDM criteria not specified. Reported by midwives at delivery. Inconsistency in methods and diagnosis	N White European, Black African, Black Caribbean South Asian Other minorities	174,320 131,201 4,927 4,698 20,086 13,408	Crude prevalence 1.0% 0.7% 1.8% 1.1% 2.0% 2.0%	Crude OR* (ref) 2.62 (1.83–3.74) 1.21 (0.72–2.02) 3.00 (2.51–3.57)	Advancing maternal age and BMI was associated with significantly higher risk for risk for GDM in South Asian and Black African women than in White European

a Figures calculated based on number of cases reported in the paper.

b Figures in subgroups not reported.

* For the BMI group: 18.5–24.9.

## Part I: Variations in adiposity, hyperglycaemia, and pre-eclampsia in pregnancy between minority groups of Asian and African descent and ethnic Europeans

### Pre-pregnant adiposity and gestational weight gain

The health risks associated with overweight and obesity are, among other factors, attributable to the adipose tissue being a potent endocrine and paracrine organ (28, 29). Adipose tissue holds metabolic functions which lead to increased levels of inflammation. Cytokine secreting macrophages may affect the insulin signalling pathway and cause increased insulin resistance (26, 30). The hormones and cytokines produced by adipose tissue are therefore important links between obesity and obesity-related complications, including the adverse outcomes seen in pregnancies (31). Obesity status is usually defined based on body mass index (BMI), with overweight at 25–29.9 kg/m^2^ and obesity at BMI≥30 kg/m^2^ (32), although this is a crude measure of adiposity, particularly in the mid-ranges of BMI (33).

Today, more women enter their pregnancies as overweight or obese. Data from the United Kingdom show that in 2007, 16% of the women entered their pregnancy as obese, and the rate of increase seems to be accelerating (14). Both pre-pregnant obesity and excessive weight gain during pregnancy are found to increase the risk for GDM and pre-eclampsia in the mother and are linked with higher post-partum weight retention (34, 35). Pre-pregnant obesity has also been indicated as an independent risk factor for adverse pregnancy outcomes for the offspring (36).

Six European studies which compared weight status in pregnant women of Asian and African descent with the majority population were identified (Table 1). Just one study had a nationally representative sample of pregnant women with register data from more than 600,000 births in the United Kingdom between 1989 and 2007 (37). Among women categorised as Black/Black British, more than 50% were either overweight or obese, and this was the only group with significantly higher odds ratio (OR) of obesity than White women in further analyses. A previous, smaller population-based study from the United Kingdom did not find the same, but had a very low number of Black women (38). The four remaining studies had maternal weight status included as baseline characteristics in cohort studies. Thus, the representativeness of the prevalence figures is likely to be variable. However, one consistent finding from these studies from the Netherlands (39), Switzerland (40), and Norway (41) was that women originating from African and Middle Eastern countries tended to enter their pregnancies with higher BMI levels than the majority population in each country.

**Table 4:** Comparison of the risk of pre-eclampsia for groups of Asian and African origin and the majority population in European countries

Author, year, (reference number)	Study design	Study population	Variable	Ethnic groups	n	Results		Comments
Khalil et al. 2012 (62)	Prospective cohort study	Pregnant women in 3 hospitals in South England, UK	Pre-eclampsia, defined affording to International Society for the Study of Hypertension in Pregnancy (ISSHP)	N Caucasian Afro-Caribbean South Asian East Asian Mixed	76,158 57,564 11,395 3,645 1,793 1,761	1,698 cases (2.3%)^a^^b^	Unadjusted OR (ref) 2.91 (2.62–3.24) 1.58 (1.28–1.95) 1.03 (0.72–1.47) 1.23 (0.89–1.72	Data collected as part of routine antenatal care. Number of cases in subgroups not reported. Significantly higher OR for pre-eclampsia in Afro-Caribbean and South Asian than Caucasian women
Bouthoorn et al. 2012 (78)	Population-based prospective cohort study	Pregnant women, The Generation R Study, Rotterdam, the Netherlands, 2002–2006	Pre-eclampsia, defined according to ISSHP	N Dutch Turkish Moroccan Cape Verde Surinamese-Creole^c^ Surinamese-Hindustan	6,215 3,886 718 534 331 232 250	120 cases (2.1%)^a^ 1.9% 1.6% 0.8% 4.2% 2.4% 3.8%	Unadjusted OR (ref) 0.83 (0.44–1.57) 0.40 (0.14–1.10) 2.22 (1.22–4.07) 1.24 (0.5–3.11) 2.04(1.00–4.13)	4–11% missing values for hypertensive pregnancy complications in ethnic groups

a Figures calculated based on number of cases reported in the paper.

b Figures in subgroups not reported.

c Results for the Surinamese, who are reported to have at least partly African (the Creoles) and Indian (the Hindustanis) ancestral origin are given in this table, but not for women from the Antilles.

All six studies found, on the other hand, that pregnant women of South and East Asian descent overall were leaner than the European population. For instance, Asian/Asian British and Chinese/other had significantly higher OR for lean (BMI <18.5) vs. ideal weight compared with White British women (37). However, these figures need to be interpreted considering studies showing substantial differences in the amount of body fat relative to BMI across ethnic groups (42), especially in Asians (43, 44), where the body fat percentage appears to be from 1–8% higher among Asians compared with Caucasians at a given BMI, sex, and age (45). The clinical relevance of the variations in body composition is supported by findings that South Asians appear to be more insulin resistant than Europeans for the same level of BMI (46). Thus, altered risks of chronic diseases may attend at different levels of BMI in different groups defined by ethnicity (44, 47); therefore, it is difficult to conclude on the true variations in adiposity between Asian pregnant women and their European counterparts. A more widespread use of the suggested lower BMI cut-off values for defining overweight (BMI≥23 kg/m^2^) and obesity (BMI≥25 kg/m^2^) among South Asians and other Asian groups should be considered in further studies (47).

Data are very limited on ethnic variations in gestational weight gain in Europe, with only two relevant studies from Switzerland identified (Table 2). Neither of the studies had ethnic variations in gestational weight gain as a main study aim. In the largest of these, both Asians and Blacks had lower weight gain than Caucasians over the whole pregnancy period (48). The other study reported somewhat higher mean net weight gains among women from Turkey, the Middle East, and Far East compared to Swiss women, and lower among women from Sri Lanka, South Asia, and Africa, but many of the subgroups had very few participants (40). A review concerning variations in and health risks of gestational weight gains (35) concludes that also American data on determinants of gestational weight gain are sparse. In one North American study, White women tended to gain excessively, while more Black women had inadequate gestational weight gain adjusted for pre-pregnant BMI and other factors (49).

**Table 5:** Comparison of differences in birth weight for groups of Asian and African origin and the majority population in European countries

Author, year, (reference number)	Study design	Study population	Variable	Ethnic groups	n	Results	Comments
Goedhart, 2008 (122)	Population-based cohort study	Singleton, live births born in Amsterdam, the Netherlands, Jan 2003–March 2004.	Birth weight	N Dutch Turkish Moroccan Ghanaian (African) Other minorities	7,118^a^ 3,859 361 629 137 2,123	Mean birth weight 3,548 g 1st gen: 3,469 g 2nd gen:3,429 1st gen: 3,527 g 2nd gen:3,419 g 3,366 g	All ethnic groups had lower mean birth weight for gestational age compared with the native Dutch.
Kelly et al. 2008 (123)	Population-based cohort study	A random sample of all births in England and Wales, 2000–2001.	Birth weight	N British White India Pakistan Bangladesh Black African Other minorities	17,769^a^ 14,068 433 687 215 327 2,339	Mean birth weight 3,416 g 3,072 g 3,110 g 3,089 g 3,343 g	Bangladeshi neonates were on average 327 g lighter than British whites. Indians 344 g, Pakistanis 306 g, and Black African 73 g lighter.
Moser et al. 2008 (124)	Population-based register study	All live births in England and Wales, UK, 2005	Birth weight	N British White India Pakistani Bangladesh Black African Other minorities	649,371^a^ 418,052 16,053 24,290 8,241 3,535 179,200	Mean birth weight 3,393 g 3,082 g 3,130 g 3,075 g 3,288 g	
Harding, 2006 (127)	Population-based register study	Hospital records from two municipalities in Lisbon, Portugal, July 2001–June 2002	Birth weight	N Native Portuguese Foreign-born African Portugal-born African	3,918 2,744 750 424	Mean birth weight 3,252 g 3,307 g 3,232 g	Term neonates of foreign-born African mothers were slightly heavier than White Portuguese neonates, but this difference disappeared after basic adjustments.
Drooger, 2005 (128)	Population -based cohort study	‘The Generation R study’ (low-risk pregnancies), Rotterdam, the Netherlands (data collection from 2002).	Estimated foetal weight (by ultrasound at GW 40)	N Native Dutch Moroccan Turkish Cape Verdean Other minorities	1,494 741 53 66 30 604	Mean birth weight 3,519 g 3,447 g 3,389 g 3,210 g	Differences not significant in Moroccan neonates or in Turkish neonates after adjustments for maternal height/weight, parity, and gender.
Harding, 2005 (125)	Population-based register study	National birth data from Portugal 1995–2002, for two ethnic groups.	Birth weight	N Portuguese African	872,463 849,595 22,463	Mean birth weight 3,303 g 3,297 g	No difference in mean birth weight in the two groups.
Vahratian, 2004 (129)	Population-based register study	Births at three public hospital prenatal clinics in Belgium, May 1994–April 1995.	Birth weight	N Native Belgians North African	1,162 808 354	Mean birth weight 3,184 g 3,338 g	Neonates born by North African immigrants were 154 g heavier than native Belgians, but this difference disappeared when adjusting for gestational age.
Harding, 2004 (126)	Population-based register study	One percent of all births in England and Wales, 1983–2000.	Birth weight	N British White UK-born Indian Foreign-born Indian UK-born Pakistani Foreign-born Pakistanis UK-born Bangladeshi Foreign-born Bangladeshi UK-born Black African Foreign-born Black African Other minorities	57,674^a^ 52,554 491 1,297 417 1,121 99 896 75 224 300	Mean birth weight 3,400 g 3,033 g 3,066 g 3,110 g 3,123 g 3,026 g 3,110 g 3,167 g 3,302 g	All groups had significantly lower mean birth weight, compared with British White neonates. After adjustments for maternal factors, mean birth weights were similar in neonates born by UK-born and foreign-born mothers within these ethnic groups.
Vangen, 2002 (130)	Population-based register study	All births in Norway from 1980–1995 for four ethnic groups.	Birth weight	N Norwegian Pakistani Vietnamese North African Other minorities	820,256 808,658 6,854 3,283 1,461	3,530 g 3,244 g 3,202 g 3,559 g	Neonates born by Pakistani mothers were 286 g lighter, Vietnamese 328 g lighter, and North African 29 g heavier than neonates born by Norwegian mothers.

a Results from women of other European and American nationalities not reported in this table.

### Gestational diabetes mellitus

In normal pregnancies, maternal immune responses occur to prevent rejection of the foetus and placenta. Maternal and placental hormones alter the carbohydrate and lipid metabolism to ensure shunting of nutrients to the foetus (26). Pregnancy can be considered as a diabetogenic and inflammatory state due to the higher levels of maternal insulin resistance, hyperlipidaemia, and fat deposition. These normal alterations are aggravated by maternal adiposity. The insulin resistance in pregnancy increases about 50–60% during pregnancy, irrespective of the pre-pregnant level (26). Thus, overweight and obese women start their pregnancy more insulin resistant compared with normal weight women and become highly insulin resistant in the second half of their pregnancy (26). This is also the case for pregnant women from East and South Asia, as they are found to be more insulin resistant compared with Western Europeans for the same level of BMI (46, 50, 51).

Pancreatic *β*-cells must compensate for the pregnancy-induced insulin resistance with increased insulin secretion. If not, hyperglycaemia may occur (52). South Asian women are reported to be less able to increase their *β*-cell function mutual to the pregnancy-induced insulin resistance compared with Western Europeans (50). Reduced *β*-cell insulin response, in the setting of insulin resistance, is also seen among Asians outside pregnancy (51).

GDM is defined as any degree of glucose intolerance with onset or first recognition during pregnancy (53, 54). The initial GDM criteria, which are in use today with only minor modifications (55), were primarily set to identify women predisposed to develop T2DM later in life (56). The WHO criteria, most commonly used in Europe, include the definition of diabetes and impaired glucose intolerance outside pregnancy (57). The International Association of Diabetes in Pregnancy Study Groups (IADPSG) recommend universal screening, and has proposed new criteria, set to identify women with increased risk of adverse foetal outcomes (54). Until now, a two-step screening procedure has mostly been used in clinical practice and research, implying that only women with definite risk factors have had an oral glucose tolerance test, which is the gold standard for a definite diagnosis of GDM.

The different methodologies, diagnostic criteria, and screening practises make comparison of prevalence figures between populations a challenge (58). GDM prevalence figures reported from Europe range from 1 to 22% (58) and will increase substantially if the IADPSG criteria are adopted (54). The HAPO study found an overall GDM prevalence of 18%, ranging from 9.3 to 26% at the different study sites, with the IADPSG criteria (59). In line with the trends for obesity and T2DM, increasing rates of GDM and undiagnosed T2DM in pregnant women attending antenatal care are observed in most parts of the world (60, 61).

In the searches, only three European studies, two from the United Kingdom and one from Norway, were identified with a main outcome to compare the risk of GDM in ethnic minority groups with the majority population (Table 3). The prevalence of GDM differs substantially in the three studies, reflecting the different screening practises and diagnostic criteria and sampling methods.

Nevertheless, within the study population, all the studies found a 2–3 higher OR for GDM in women of South Asian origin (41, 62, 63). The grouping of the ethnic minority population varies between the studies, but also Black African (63), East Asian and Afro-Caribbean (62), and Middle Eastern (41) women had significantly higher OR for GDM in the three studies. The study based on the large population in London also points to that women of Black African and South Asian origin had higher risk of GDM at a younger age compared with White British women (63). Similar ethnic differences and interactions with age have been found in Australia (64), United States (65), and Canada (66). Furthermore, women with South Asian origin developed GDM at a lower BMI compared with other ethnic groups (63), as also confirmed elsewhere (67–69). All studies conclude that a BMI cut-off of ≥25 kg/m^2^ might not identify Asian women at risk for GDM. In addition to adiposity and age, family history and low socioeconomic status partly explain the increased risk for GDM in ethnic minority groups (41, 61, 70).

Another important aspect relevant for ethnic differences is that a two- to three-fold higher prevalence of GDM is reported when applying the new IADPSG criteria rather than the WHO criteria, mainly due to a lower cut-off value for fasting plasma glucose (41, 71, 72). The study from Oslo also showed that compared with the WHO criteria, the GDM prevalence increased 2.8 times in the South Asians, compared with 2.2 times in Western Europeans when applying the IADPSG criteria (41). In other studies, women with Arab and South Asian origin have more abnormal fasting plasma glucose values (71). However, the increase in the GDM prevalence when applying the IADPSG criteria differed in women from East and South Asia, as a pattern with relatively lower fasting values and higher 2-h glucose values was more prevalent in the East Asian women (41). This is in line with findings from the HAPO study where women from East Asia were less likely to be diagnosed with GDM with the fasting glucose value compared with women from other study sites (59). Dissimilarities in how the IADPSG criteria apply to different ethnic groups may shed light on the possible underlying etiologic variations in glucose metabolism.

### Pre-eclampsia

Pre-eclampsia, defined by the International Society for the Study of Hypertension in Pregnancy as blood pressure ≥140/90 mmHg and 24-h proteinuria ≥0.3 g, is a multisystem disorder characterised by abnormal vascular response to placentation (73). Pre-eclampsia is the leading cause of maternal mortality globally (74) and may seriously affect foetal growth. Delivery is the only curative treatment. The incidence ranges from 3 to 7% for nulliparous and 1 to 3% for multiparas (73), with slightly increasing rates in recent years (74). Early pre-eclampsia (<37 weeks of gestation) is most serious for the mother and the foetus, and is more likely to recur in later pregnancies (74). The risk factors for pre-eclampsia may be divided in two subgroups: those related to immunogenetic factors and those related to maternal disease (75). Women with pre-existing hypertension, diabetes, obesity, GDM or a close relative with pre-eclampsia or early CVD are at increased risk (76, 77).

Only two European studies exploring ethnic differences in pre-eclampsia were found (Table 4). In the largest study from United Kingdom, women of Afro-Caribbean origin had a three-fold higher OR compared with the Caucasians, and South Asians had an OR of 1.6 (62). In a study from the Netherlands, women from Cape Verde and Creoles from Suriname (with at least partly African origin) had increased risk (78). Studies from the United States have consistently found that women of African ancestral origin have the highest risk for pre-eclampsia (75, 79). However, East Asian women may have lower risk than White women (75).

## Part II: Health consequences of excessive weight gain, gestational diabetes, and pre-eclampsia for the mother after pregnancy – are ethnic differences observed in Europe?

### Risk of future obesity

Pregnancy has been considered a critical period for development of overweight and obesity. A significant proportion of the gestational weight gain is lost at delivery, and the natural course is to lose most of the remaining weight within 12 months post-partum (80). However, women with excessive gestational weight gains have increased risk of post-partum weight retention (34, 81). Only one European study reporting post-partum weight retention across ethnic groups was found (Table 2). In this Dutch study, 52% of the Turkish mothers had retained more than 5 kg, 3–5 months post-partum, compared with 26% of the Dutch, and this difference was significant in further analyses (82). Parity, that is, the number of times a woman has given birth, has been suggested as a risk factor for development of obesity, indicating progressive weight gains in mothers with many children. Such mechanism would be of importance to groups with high fertility rates. However, recent systematic reviews contest whether parity is an independent risk factor for obesity development in general (80). North American studies have also explored the role of parity with specific relevance to ethnic differences in post-partum weight retention and find inconsistent relationships (83–87). Thus, weight retention patterns in ethnic minority groups in Europe and their determinants still need to be explored.

Breastfeeding has been considered to have a beneficial effect on post-partum weight loss. Two large studies, one from Denmark (88) and one among racially diverse women in the United States (89), show a positive effect of breastfeeding on long-term post-partum weight loss compared with formula feeding, with stronger effects with more intensive and longer duration of breastfeeding. However, the WHO Multicentre Growth Reference Study found that although breastfeeding mothers in Norway, USA, Brazil, and India followed the expected trend (sustained loss the first year, plateaus or slight upward shifts the second), breastfeeding mothers in Oman lost little weight and in Ghana they tended to gain weight (90). This study points to that ethno-cultural practices related to the mothers’ food-intake and physical activity pattern seem to attenuate the effect of breastfeeding on weight retention. Although the overall rates of breastfeeding differ between European countries, most studies find that ethnic minority women breastfeed longer than the majority population (91) with some exceptions (92). However, the United Kingdom Infant Feeding Survey showed that for every additional 5 years spent in the United Kingdom, immigrant mothers were 5% less likely to breastfeed for at least 4 months (93) and the prevalence of breastfeeding is lower than in their countries of origin (94).

A systematic review points to obese mothers being less likely to breastfeed compared with normal weight mothers (95), which may reinforce a tendency to gain weight, although one study from the United States indicated that this association could vary between ethnic groups (96). Little is known about possible associations between maternal weight status, breastfeeding problems and ethnic variations in Europe.

### Development of type 2 diabetes in women with previous gestational diabetes

The increasing trend for GDM globally, and the relatively higher susceptibility of many ethnic minority women in Europe today, is worrisome. GDM may reflect either a pre-existing, undiagnosed T2DM or a pregnancy-induced glucose-intolerant state with a high risk of future T2DM (97). Therefore, most clinical guidelines recommend a sustained screening program after a pregnancy complicated by GDM (98), as T2DM may be prevented or postponed in these women (99). The gradual fall in *β*-cell function and progression into T2DM is influenced by when in the pregnancy GDM developed, insulin needs, obesity, and ethnicity; though the relation with ethnicity is complex and seems to differ between studies (98, 100). Recurrence rates of GDM in subsequent pregnancies have been found to vary between 30 and 84%, with higher rates in minority groups compared with White populations (97). However, those who did not develop GDM in a subsequent pregnancy had a reduced risk of T2DM (101).

We found only one small European study exploring ethnic differences in conversion rates (102). A recent review, covering the early post-partum period, found overt T2DM in 1.2–4.5% of the women, but impaired glucose tolerance or impaired fasting glucose in as many as 12–36% (98). South Asians had higher rates of both screening and abnormal results. Generally, differences in reported incidence rates of T2DM may reflect differences in screening procedures and attendance (98), and the rates of abnormalities are probably underestimates, as several studies only used fasting glucose (103). The first review also covering a longer follow-up, included 28 studies, five were from White populations in Europe; the majority were from the United States (100). The cumulative incidence of T2DM after the index pregnancy with GDM was found to range as much as from 2.6 to 70%, dependant on length of follow-up, and with the highest incidence found in ethnic minorities (100). The incidence increased rapidly the five first years to about 50% overall, and then more slowly after 10 years.

A later systematic review of 20 studies including GDM women and a control group, found that women with previous GDM had a seven-fold increased relative risk of developing T2DM, with a slight increase in risk in recent years (104). When it comes to conversion rates in recent studies, a large Canadian register study found that women who had GDM reached an incidence of T2DM of 16% after 5 years (105). The meta-analysis revealed that the relative risk was generally consistent for subgroups (age, BMI, diagnostic criteria, study size), including ethnicity (104). Eight studies were from Europe; the only one exploring ethnic differences in conversion to T2DM found that women of North African origin had higher conversion rates than the majority population (102). Most of the other studies did not provide comparisons of ethnic groups relevant for Europe. Some recently published studies indicate that women of Asian (106, 107) or African origin (108) may be at an even higher risk of progression to T2DM than White women. Nevertheless, clinicians in Europe today need to be aware of the high prevalence of GDM and the substantially increased risk of T2DM regardless of ethnicity for women with pregnancies complicated by GDM.

### CVD in women with previous gestational diabetes and pre-eclampsia

In recent years, associations between GDM or pre-eclampsia and later CVD are increasingly recognised (20, 21). Women with previous GDM may display early signs of increased CVD risk through higher values for endothelial dysfunction, inflammatory markers, and metabolic abnormalities than controls (109). Furthermore, T2DM negates the protective effect of being a female on the risk of CVD (110). Little is known about ethnic differences in the risk of future CVD in women with previous GDM, as this is a rather new issue which has to be studied in multi-ethnic cohorts with long-term follow-up. In a large Canadian population-based cohort, not stratified for ethnicity, previous GDM gave a 70% increased risk of developing CVD after 12 years of follow-up (111). After adjusting for T2DM, the risk was attenuated, indicating that much of the excess CVD risk was mediated by the development of T2DM. Therefore, prevention of T2DM in women with GDM seems indicated also with respect to CVD (99, 111).

Furthermore, women who develop pre-eclampsia seem to be at increased risk for CVD later in life (20). Obesity and concomitant hyperlipidaemia, hypertension, and other disorders associated with pre-existing endothelial dysfunction, such as the metabolic syndrome and T2DM, are factors shared by women at risk of both pre-eclampsia and CVD (112). Endothelial dysfunction has been observed at 23 weeks of gestation in women who later developed pre-eclampsia, during pre-eclampsia, and 3 months after pre-eclampsia has resolved (113, 114). Little is known about ethnic differences in susceptibility for CVD in women with pre-eclampsia. In a meta-analysis, women with a history of pre-eclampsia had a four-fold increased risk for hypertension, a two-fold increased risk for ischemic heart disease, stroke, and deep venous thrombosis, and 1.5-times higher all-cause mortality (115). The doubled risk of later CVD seemed to persist more than 20 years after the index pregnancy (115). Associations between pre-eclampsia and future CVD seem independent of other known risk factors. Early onset pre-eclampsia gave the greatest risk of future CVD with a seven-fold increase (115). For the outcome ischemic heart disease, five of eight studies were European, and only one study from the United Kingdom included a population of mixed ethnicity (116), but results were not analysed according to ethnic origin. The excess risk of pre-eclampsia in women of African ancestral origin is likely contributing to their excess risk of hypertension and CVD (115).

## Part III: Ethnic differences in prenatal development and possible effects on future risk of obesity, diabetes, and CVD

### Developmental origin of health and disease

There is now strong evidence supporting that early development plays a central role in determining an individual's risk of later adult disease (117). In short, this involves mechanisms of developmental plasticity, including epigenetic processes enabling the development of a phenotype appropriate for the environment in which the offspring is predicted to live (118). The phenotypic characteristics may relate to metabolic control, skeletal muscle fibre, cardiomyocyte, and nephron numbers and control systems such as appetite, stress responses, and timing of puberty. It is thought that mechanisms exist, mediated through placental function, by which the mother modulates foetal growth to be appropriate to her stature, pre-pregnant condition, and nutritional status (119). A poor intrauterine environment may predispose the offspring to favour brain growth while restricting growth of internal organs, bone and muscle, and fat deposition in the third trimester in anticipation of a poor post-natal environment (119).

Although the adaptations seem advantageous favouring short time survival, fitness, and reproduction, they may turn out to be inappropriate in the actual environment the offspring is born into – a term called *developmental mismatch* (117). This mismatch may result in long-term increased susceptibility to non-communicable diseases, such as obesity, T2DM, and CVD (120), as observed in ethnic minorities in Europe. The mismatch may arise through unbalanced diet or body composition of the mother or a change in environment and lifestyle factors between generations. This may particularly be the case when there are rapid rural to urban transitions, as in developing countries, and for immigrants from low- and middle-income countries to Europe, where the mother developed in a very different environment from that where she is pregnant (119).

A rather new developmental pathway to obesity is hyper-nutrition during foetal life, also called the *foetal overnutrition pathway* (120). This may create a ‘vicious cycle’ where the increasing prevalence of obesity and T2DM in the mothers endorses obesity in later generations (121). The foetal overnutrition pathway may be particularly important in Europe and other Western societies today, irrespective of ethnic background. These early life developmental factors are not thought to cause later non-communicable diseases, but may influence such risks through different non-mutually exclusive pathways, especially in a later obesogenic environment (117).

### Neonatal size and body composition

Birth weight is the most accessible and described measure reflecting foetal growth. We identified nine European studies comparing birth weight in different ethnic groups (Table 5). Generally, birth weight was lower for most groups with maternal origin from low-income countries than for the majority population, with babies of Asian descent, particularly South Asian, being the smallest (122–130). A few studies found that neonates of North African and Sub-Sahara African descent had similar or even slightly higher birth weight than the majority population (125, 129, 130). Ethnic differences in intrauterine growth seem to become more pronounced towards term (128) and seem to persist also for neonates of the second generation of mothers (126, 127). In some studies, parental (131) and socioeconomic factors (123) may partly explain ethnic differences in birth weight.

Assessment of neonatal body composition, that is, the description of lean and fat mass, gives a better indication of growth relative to the *in utero* environment than birth weight alone (26). No studies from Europe were identified, but large differences in neonatal body composition were found when comparing neonates born in the United Kingdom, India, Congo, Finland, Sri Lanka, China, Nigeria, and Jamaica (132). Indian neonates from a poor rural district had small abdominal circumference and low muscle mass, but relatively preserved body fat, compared with babies born in Southampton, United Kingdom (133, 134), also called ‘the thin-fat-phenotype’. This is proposed to represent a predisposition to T2DM present at birth (133, 134). Differences in neonatal body composition between groups with European and African ancestry have been found in the United States (135), mostly due to a relative reduction in lean mass. The patterns and timing of tissue growth also seem to differ between these groups (136).

Infants of mothers with obesity or hyperglycaemia are heavier at birth than those of normal weight or normoglycaemic mothers, mostly related to an increase in fat mass (137). In offspring of women with obesity and GDM, increased maternal pre-pregnant insulin resistance is the strongest predictor for neonatal fat mass (138). In line with this, the large HAPO study, covering several ethnic groups, showed an independent continuous and graded relationship between maternal glycaemia and maternal BMI, and foetal outcomes such as birth weight above the 90th percentile and neonatal adiposity (139). When assessed together, both maternal hyperglycaemia and obesity were independently associated with increased adiposity in the newborn (140), and their combination had a greater impact than either one alone. However, recent studies have found that the influence of maternal obesity and GDM seemed to be stronger in ethnic minority groups than in groups with European ancestral origin (141, 142) and that the combined effects were particularly strong in these groups.

Pre-eclampsia is, on the other hand, associated with an increased risk of intrauterine growth restriction (143), often characterised by a relative sparing of head and skeletal growth, and relatively more reduced abdominal size and subcutaneous fat mass. In line with the observed susceptibility for pre-eclampsia in women of African-Caribbean origin, their offspring seem to be at increased risk of growth restriction and other adverse outcomes related to pre-eclampsia (62).

### Early origins of childhood and adult obesity

Although many factors will contribute to obesity development over the life course, both a restricted and an excessive *in utero* environment appear to have an independent effect on later risk of obesity and related chronic diseases (144). In a large systematic review, maternal BMI, early rapid growth, early adiposity rebound, childhood obesity, and low paternal employment status were the factors most consistently associated with adult obesity (145). However, studies exploring the associations between low and high birth weight and subsequent adult obesity showed mixed results. This may partly be related to the fact that birth weight is a crude indicator of the uterine environment. Prenatal influences on the development of the liver, pancreas, and endothelial cells, and an altered body composition may be more important in explaining differences in obesity and metabolic risk than birth weight (146). We found no longitudinal studies in ethnic minorities regarding early origin of adiposity.

Also early feeding practices influence infant growth and subsequent risk of adult obesity. After the first 2–3 months, breastfed infants grow at a slower rate than those who are formula fed (147). In the Dutch ABCD-study, the growth rate from 1 to 6 months was higher in infants of African, Turkish, and Moroccan descent than in the native Dutch. Breastfeeding duration and exclusive breastfeeding at 4 months were associated with slower growth in all ethnic groups. Lower rates of breastfeeding partly explained the increased growth rate in infants of African descent (148). Additionally, weight gain during the first 6 months of life explained a large proportion of the increased prevalence of overweight in Turkish and Moroccan 2-year olds (149).

Present evidence suggests that breastfeeding (150–155), particularly prolonged breastfeeding (156–159), may exert a modest protective effect on childhood and adolescent obesity, although some of the evidence is contradictory (150). On the other hand, recent studies including siblings suggest that breastfeeding selectively protects against extremes of childhood size (152, 153). Most studies have included only mothers and children of European descent. Suggested mechanisms are hormones in breast milk, such as leptin and adiponectin, which may determine appetite signalling (160). The higher protein concentration in infant formula compared with human milk (161) and the ability of breastfed infants to self-regulate their energy intake are other possible mechanisms (162).

### Early origins of type 2 diabetes

Many studies have replicated the first report showing a graded inverse association between birth weight and risk of T2DM (163), particularly if followed by high childhood or adult weight gains (146). A systematic review and meta-analysis based on 31 populations, including six from Asia (164) found that the pooled age- and sex-adjusted OR for T2DM was reduced by 25% per kilogram increased birth weight, after exclusion of two studies from native North Americans, demonstrating a U-shaped association. Adjustment for current BMI slightly strengthened the association, but socioeconomic status did not materially affect the estimate (164).

Although middle-aged and older populations studied in the later 20th century have strong inverse associations between birth weight and T2DM, it remains uncertain whether this pattern will persist (164). The U-shaped curve observed in Native Americans, as well as younger cohorts, indicates that high birth weights may also confer a risk. This pattern may be more common as maternal obesity and hyperglycaemia become more prevalent (140). Breastfed infants seem to have a modest reduction in risk of T2DM in later life compared with formula fed infants, as well as marginally lower insulin levels (165). Similar effects have been observed across different population groups.

A recent phenomenon is that T2DM is now diagnosed even in children and adolescents, strongly related to obesity, parental T2DM, and ethnicity (166). In the United States, T2DM constitutes nearly half of new cases of diabetes diagnosed in adolescence, compared with only 1–2% of all diabetes cases in youth in Europe (167). Few European studies about T2DM in children and adolescents have been published, but several report T2DM precursors. A systematic review of British studies found no consistent differences in obesity prevalence among children of different ethnic backgrounds (168). However, a large cross-sectional study of British school children found higher levels of adiposity in several ethnic minority groups, especially those of South Asian origin, compared with White children (169). Furthermore, the metabolic profiles of South Asian children were more insulinogenic and atherogenic compared with White British children, and some T2DM precursors were also elevated in children of Black African-Caribbean descent (170). Milder dysglycaemia and impaired glucose tolerance have been found in 25% of children with a severe degree of obesity, irrespective of ethnicity (171). Factors associated with more rapid progression are marked weight gain and profound insulin resistance. Thus, possible rapid conversion to T2DM in high risk individuals from the most susceptible ethnic minority groups should not be ignored.

### Early origins of CVD

Associations between birth weight and later CVD have been found in numerous studies (24, 25). A recent review and meta-analyses of 22 studies, mostly from Europe, found that 1 kg higher birth weight was associated with a 12% lower risk of CVD mortality, and a 6% reduced risk of all-cause mortality (172).

A higher incidence of CVD, appearing at a younger age, has been seen in several ethnic minority groups compared with ethnic Europeans (173). We can only speculate if the lower birth weight and the higher proportion of neonates in the lowest birth weight categories observed in many ethnic groups may be a potential explanatory factor for the observed differences in CVD risk. The higher prevalence of pre-eclampsia in women of African origin (62) could also possibly contribute to a higher incidence of CVD through restricted foetal growth.

## Conclusions

The health profiles of ethnic minority women of Asian and African origin in Europe are still relatively poorly documented. Summed up, pre-pregnant BMI appears to be heterogeneous between groups, but the studies point to higher levels of obesity in women of African, possibly also Middle Eastern descent, while women from South and East Asia tend to have lower BMI than the majority populations. Differences in gestational weight gain and post-partum weight retention are hardly documented in European immigrant populations, but variations are likely. However, ethnic variations in adiposity at the same level of BMI may be of importance to interpret the true health risks in these women. This is shown especially in the significantly higher risk of GDM in many groups, particularly those of South Asian ancestry, even at lower BMI levels.

A GDM pregnancy implies a substantially increased risk of future T2DM, although ethnic differences in conversion rates may exist. GDM may also imply a slight predisposition to later CVD. African origin is associated with increased risk of pre-eclampsia compared with women of European descent, and pre-eclampsia may influence later development of CVD.

Regarding the neonates of women descending from Asian and African countries, the birth weights are generally lower than in the majority population, a tendency that seems to persist, at least over some generations. A few studies find higher birth weights in babies of mothers of African descent. The small neonatal size in ethnic minority groups is most likely, at least in part, due to persistent maternal constraints on foetal growth and can probably be regarded as mismatched when exposed to the current obesogenic environment. Evidence is mounting that low birth weight increases the risk of later obesity, T2DM, and CVD. Furthermore, neonates exposed to maternal obesity and GDM may carry an increased risk of the same conditions linked to foetal overgrowth.

This review draws upon the awareness that pregnancy can be considered a natural stress test that can reveal the future risk of T2DM and CVD in women (20). The increasing trend in the prevalence of GDM and the higher risk found in many ethnic minority groups are of great concern, and further application of the new IADPSG criteria for diagnosis may lead to further unfolding of the problem. As maternal obesity also carries independent risks of adverse outcomes both in the mother and the offspring, this condition also deserves more attention.

Although genes obviously play a role for individual susceptibility, research at the forefront is now addressing the ‘soft inheritance’ and epigenetic mechanisms, which are highly relevant for the observed ethnic variations in health and disease (117). The combined effects of early and later life exposures, against a genetic predisposition, are needed to understand the ethnic differences in prevalence of T2DM and its underlying metabolic disturbances (174, 175).

### Implications

The pregnancy and post-partum period is an important, but presently underused, window of opportunity for prevention of future non-communicable diseases both in the women herself and for the next generation (118). New public health initiatives need to focus more on early life interventions as interventions in adulthood have shown limited results (118). The widespread coverage of antenatal care in Europe has considerable potential to promote awareness about healthy diets, physical activity, and appropriate weight gains in pregnant women. Pregnant ethnic minority women, and probably most women today, should be screened for GDM. However, the current WHO manual for the antenatal care has little focus on the long-term prevention of non-communicable diseases in the mother and her offspring (176).

In the post-partum period, women with pregnancies complicated by GDM should be screened by an oral glucose tolerance test to identify overt T2DM as well as milder forms of dysglycaemia, where development of T2DM may be prevented or delayed by lifestyle interventions (99). Other high risk women, such as those with pre-eclampsia, and with excessive weight gain in the previous pregnancy, should be offered individual counselling related to their future risk of obesity, T2DM, and CVD. As breastfeeding may be associated with a decreased risk of obesity and adult disease both for the mother and baby (88, 89, 154, 165), current efforts to promote breastfeeding should be strengthened, not least for ethnic minority groups.

However, there is still no uniform strategy for how excessive weight gain during pregnancy and adverse pregnancy outcomes best can be prevented on a population level (177–182), but it is clear that interventions targeting ethnic minority groups need to be culturally sensitive and tailored to their specific needs (178). Both research into development of culturally sensitive and evidence-based interventions targeting the most susceptible ethnic groups during pregnancy and post-partum and efforts to implement such strategies at a larger scale are highly needed. This review also revealed that only few studies address ethnic differences in maternal and perinatal health in Europe. This should be a prioritised area for research in the future, recognising the importance of early life for later health and disease.

## Appendix 1

**Table:** Search terms with regard to ethnic differences in the pre-pregnant BMI, gestational weight gain, post-partum weight retention, prevalence of gestational diabetes mellitus, prevalence of pre-eclampsia, and birth weight and body composition of neonates.

	Search terms			
	
Table No, outcome	Main area	Demarcations		Joint search string
1: pre-pregnant weight	Body constitution	(Pregnant OR pregnancy)	Europe	(Human migration OR Africa OR Asia OR ethnic OR ethnicity OR race OR racial OR immigrant OR immigrants OR minority OR minorities OR ethnology)
2: gestational weight gain	Weight gain	(Pregnant OR pregnancy)	Europe	
2: post-partum weight retention	Postpartum weight retention	(Pregnant OR pregnancy)	Europe	
3: gestational diabetes mellitus	Diabetes, gestational	(Pregnant OR pregnancy)	Europe	
4: pre-eclampsia	Pre-eclampsia	(Pregnant OR pregnancy)	Europe	
5: birth weight	(Birth weight OR birth weight OR body composition OR fat OR lean OR thin)	(Newborn OR infant OR neonatal)	Europe	(Ethnology OR ethnic OR ethnicity OR race OR racial OR minorities OR groups, minority)

Filters: 10 years, English.

## References

[1] Fry J, Finley W (2005). The prevalence and costs of obesity in the EU. Proc Nutr Soc.

[2] WHO Global Infobase (2011). https://apps.who.int/infobase/.

[3] Logue J, Sattar N (2011). Childhood obesity: a ticking time bomb for cardiovascular disease?. Clin Pharmacol Ther.

[4] Jenum A, Diep L, Holmboe-Ottesen G, Holme I, Kumar B, Birkeland K (2012). Diabetes susceptibility in ethnic minority groups from Turkey, Vietnam, Sri Lanka and Pakistan compared with Norwegians – the association with adiposity is strongest for ethnic minority women. BMC Public Health.

[5] Gholap N, Davies M, Patel K, Sattar N, Khunti K (2011). Type 2 diabetes and cardiovascular disease in South Asians. Prim Care Diabetes.

[6] Wandell PE, Carlsson A, Steiner KH (2010). Prevalence of diabetes among immigrants in the Nordic countries. Curr Diabetes Rev.

[7] Tran A, Diep L, Cooper J, Claudi T, Straand J, Birkeland K (2010). Quality of care for patients with type 2 diabetes in general practice according to patients’ ethnic background: a cross-sectional study from Oslo, Norway. BMC Health Serv Res.

[8] Chaturvedi N, Fuller JH (1996). Ethnic differences in mortality from cardiovascular disease in the UK: do they persist in people with diabetes?. J Epidemiol Community Health.

[9] Vandenheede H, Deboosere P, Stirbu I, Agyemang C, Harding S, Juel K (2012). Migrant mortality from diabetes mellitus across Europe: the importance of socio-economic change. Eur J Epidemiol.

[10] Lanting LC, Joung IMA, Mackenbach JP, Lamberts SWJ, Bootsma AH (2005). Ethnic differences in mortality, end-stage complications, and quality of care among diabetic patients. Diabetes Care.

[11] Bhopal RS (2013). A four-stage model explaining the higher risk of type 2 diabetes mellitus in South Asians compared with European populations. Diabet Med.

[12] Holmboe-Ottesen G, Wandel M (2012). Changes in dietary habits after migration and consequences for health: a focus on South Asians in Europe. Food Nutr Res.

[13] Gilbert PA, Khokhar S (2008). Changing dietary habits of ethnic groups in Europe and implications for health. Nutr Rev.

[14] Malmusi D, Borrell C, Benach J (2010). Migration-related health inequalities: showing the complex interactions between gender, social class and place of origin. Soc Sci Med.

[15] Nazroo JY (2001). South Asian people and heart disease: an assessment of the importance of socioeconomic position. Ethn Dis.

[16] Nazroo JY, Smith GD, Macbeth HM, Shetty PS (2001). The contribution of socio-economic position to health differentials between ethnic groups: evidence from the United States and Britain. Health and ethnicity. Society for the Study of Human Biology symposium series.

[17] Karlsen S, Nazroo JY (2002). Relation between racial discrimination, social class, and health among ethnic minority groups. Am J Public Health.

[18] Smith GD, Chaturvedi N, Harding S, Nazroo J, Williams R (2000). Ethnic inequalities in health: a review of UK epidemiological evidence. Crit Public Health.

[19] Vasileva K (2011). Population and social conditions: 6.5% of the EU population are foreigners and 9.4% are born abroad. Statistics in focus, 34/2011.

[20] Bilhartz TD, Bilhartz PA, Bilhartz TN, Bilhartz RD (2011). Making use of a natural stress test: pregnancy and cardiovascular risk. J Womens Health.

[21] Piña IL (2011). Cardiovascular disease in women: challenge of the middle years. Cardiol Rev.

[22] Temple R, Murphy H (2010). Type 2 diabetes in pregnancy – an increasing problem. Best Pract Res Clin Endocrinol Metab.

[23] Balsells M, García-Patterson A, Gich I, Corcoy R (2009). Maternal and fetal outcome in women with type 2 versus type 1 diabetes mellitus: a systematic review and metaanalysis. J Clin Endocrinol Metab.

[24] Barker DJ (1998). In utero programming of chronic disease. Clin Sci (Lond).

[25] Gluckman PD, Hanson MA, Pinal C (2005). The developmental origins of adult disease. Matern Child Nutr.

[26] Catalano PM (2010). Obesity, insulin resistance, and pregnancy outcome. Reproduction.

[27] Bhopal RS (2007). Ethnicity, race, and health in multicultural societies: foundations for better epidemiology, public health, and health care.

[28] Gnacinska M, Malgorzewicz S, Stojek M, Lysiak-Szydlowska W, Sworczak K (2009). Role of adipokines in complications related to obesity: a review. Adv Med Sci.

[29] Maury E, Brichard SM (2010). Adipokine dysregulation, adipose tissue inflammation and metabolic syndrome. Mol Cell Endocrinol.

[30] Catalano PM, Hauguel-De Mouzon S (2011). Is it time to revisit the Pedersen hypothesis in the face of the obesity epidemic?. Am J Obstet Gynecol.

[31] Mitchell M, Armstrong DT, Robker RL, Norman RJ (2005). Adipokines: implications for female fertility and obesity. Reproduction.

[32] WHO Consultation (2000). Obesity: preventing and managing the global epidemic. World Health Organ Tech Rep Ser.

[33] Rothman KJ (2008). BMI-related errors in the measurement of obesity. Int J Obes.

[34] Siega-Riz AM, Herring AH, Carrier K, Evenson KR, Dole N, Deierlein A (2010). Sociodemographic, perinatal, behavioral, and psychosocial predictors of weight retention at 3 and 12 months postpartum. Obesity.

[35] Rasmussen KM, Yaktine AL, Institute of Medicine (US) and National Research Council (US) (2009). Committee to reexamine IOM pregnancy weight guidelines. Weight gain during pregnancy: reexamining the guidelines.

[36] The Hyperglycaemia and Adverse Pregnancy Outcome Study group (2010). Hyperglycaemia and Adverse Pregnancy Outcome (HAPO) Study: associations with maternal body mass index. BJOG.

[37] Heslehurst N, Rankin J, Wilkinson JR, Summerbell CD (2010). A nationally representative study of maternal obesity in England, UK: trends in incidence and demographic inequalities in 619 323 births, 1989–2007. Int J Obes.

[38] Heslehurst N, Ells LJ, Simpson H, Batterham A, Wilkinson J, Summerbell CD (2007). Trends in maternal obesity incidence rates, demographic predictors, and health inequalities in 36 821 women over a 15-year period. BJOG.

[39] Djelantik A, Kunst AE, van der Wal MF, Smit HA, Vrijkotte TGM (2012). Contribution of overweight and obesity to the occurrence of adverse pregnancy outcomes in a multi-ethnic cohort: population attributive fractions for Amsterdam. BJOG.

[40] Loetscher KC, Selvin S, Zimmermann R, Abrams B (2007). Ethnic-cultural background, maternal body size and pregnancy outcomes in a diverse Swiss cohort. Women Health.

[41] Jenum AK, Morkrid K, Sletner L, Vangen S, Torper JL, Nakstad B (2012). Impact of ethnicity on gestational diabetes identified with the WHO and the modified International Association of Diabetes and Pregnancy Study Groups criteria: a population-based cohort study. Eur J Endocrinol.

[42] Dehghan M, Merchant A (2008). Is bioelectrical impedance accurate for use in large epidemiological studies?. Nutr J.

[43] Deurenberg P, Deurenberg-Yap M, Guricci S (2002). Asians are different from Caucasians and from each other in their body mass index/body fat per cent relationship. Obes Rev.

[44] IASO, WHO, IOTF (2000). The Asia-Pacific perspective: redefining obesity and its treatment.

[45] Wulan SN, Westerterp KR, Plasqui G (2010). Ethnic differences in body composition and the associated metabolic profile: a comparative study between Asians and Caucasians. Maturitas.

[46] Misra A, Khurana L (2011). Obesity-related non-communicable diseases: South Asians vs White Caucasians. Int J Obes.

[47] WHO Expert Consultation (2004). Appropriate body-mass index for Asian populations and its implications for policy and intervention strategies. Lancet.

[48] Ochsenbein-Kolble N, Roos M, Gasser T, Zimmermann R (2007). Cross-sectional study of weight gain and increase in BMI throughout pregnancy. Eur J Obstet Gynecol Reprod Biol.

[49] Chu SY, Callaghan WM, Bish CL, D'Angelo D (2009). Gestational weight gain by body mass index among US women delivering live births, 2004–2005: fueling future obesity. Am J Obstet Gynecol.

[50] Morkrid K, Jenum AK, Sletner L, Vardal MH, Waage CW, Nakstad B (2012). Failure to increase insulin secretory capacity during pregnancy-induced insulin resistance is associated with ethnicity and gestational diabetes. Eur J Endocrinol.

[51] Golden SH, Brown A, Cauley JA, Chin MH, Gary-Webb TL, Kim C (2012). Health disparities in endocrine disorders: biological, clinical, and nonclinical factors – an Endocrine Society scientific statement. J Clin Endocrinol Metab.

[52] Buchanan TA, Xiang A, Kjos SL, Watanabe R (2007). What is gestational diabetes?. Diabetes Care.

[53] Metzger BE, Buchanan TA, Coustan DR, de Leiva A, Dunger DB, Hadden DR (2007). Summary and recommendations of the Fifth International Workshop-Conference on Gestational Diabetes Mellitus. Diabetes Care.

[54] Metzger BE, Gabbe SG, Persson B, Buchanan TA, Catalano PA, Damm P (2010). International association of diabetes and pregnancy study groups recommendations on the diagnosis and classification of hyperglycemia in pregnancy. Diabetes Care.

[55] Committee opinion no. 504: screening and diagnosis of gestational diabetes mellitus (2011). Obstet Gynecol.

[56] O'Sullivan JB, Gellis SS, Dandrow RV, Tenney BO (1966). The potential diabetic and her treatment in pregnancy. Obstet Gynecol.

[57] Alberti KG, Zimmet PZ (1998). Definition, diagnosis and classification of diabetes mellitus and its complications. Part 1: diagnosis and classification of diabetes mellitus provisional report of a WHO consultation. Diabet Med.

[58] Buckley BS, Harreiter J, Damm P, Corcoy R, Chico A, Simmons D (2012). Gestational diabetes mellitus in Europe: prevalence, current screening practice and barriers to screening. Diabet Med.

[59] Sacks DA, Hadden DR, Maresh M, Deerochanawong C, Dyer AR, Metzger BE (2012). Frequency of gestational diabetes mellitus at collaborating centers based on IADPSG consensus panel-recommended criteria: the Hyperglycemia and Adverse Pregnancy Outcome (HAPO) Study. Diabetes Care.

[60] Ferrara A (2007). Increasing prevalence of gestational diabetes mellitus: a public health perspective. Diabetes Care.

[61] Anna V, van der Ploeg HP, Cheung NW, Huxley RR, Bauman AE (2008). Sociodemographic correlates of the increasing trend in prevalence of gestational diabetes mellitus in a large population of women between 1995 and 2005. Diabetes Care.

[62] Khalil A, Rezende J, Akolekar R, Syngelaki A, Nicolaides KH (2012). Maternal racial origin and adverse pregnancy outcomes: a cohort study. Ultrasound Obstet Gynecol.

[63] Makgoba M, Savvidou MD, Steer PJ (2012). An analysis of the interrelationship between maternal age, body mass index and racial origin in the development of gestational diabetes mellitus. BJOG.

[64] Carolan M, Davey MA, Biro MA, Kealy M (2012). Maternal age, ethnicity and gestational diabetes mellitus. Midwifery.

[65] Hedderson MM, Darbinian JA, Ferrara A (2010). Disparities in the risk of gestational diabetes by race-ethnicity and country of birth. Paediatr Perinat Epidemiol.

[66] Mukerji G, Chiu M, Shah BR (2012). Impact of gestational diabetes on the risk of diabetes following pregnancy among Chinese and South Asian women. Diabetologia.

[67] Hedderson M, Ehrlich S, Sridhar S, Darbinian J, Moore S, Ferrara A (2012). Racial/ethnic disparities in the prevalence of gestational diabetes by BMI. Diabetes Care.

[68] Wong VW (2012). Gestational diabetes mellitus in five ethnic groups: a comparison of their clinical characteristics. Diabet Med.

[69] Farah N, Murphy M, Ramphul M, O'Connor N, Kennelly MM, Turner MJ (2011). Comparison in maternal body composition between Caucasian Irish and Indian women. J Obstet Gynaecol.

[70] Link CL, McKinlay JB (2009). Disparities in the prevalence of diabetes: is it race/ethnicity or socioeconomic status? Results from the Boston Area Community Health (BACH) survey. Ethn Dis.

[71] Agarwal MM, Dhatt GS, Shah SM (2010). Gestational diabetes mellitus: simplifying the international association of diabetes and pregnancy diagnostic algorithm using fasting plasma glucose. Diabetes Care.

[72] Jiwani A, Marseille E, Lohse N, Damm P, Hod M, Kahn JG (2012). Gestational diabetes mellitus: results from a survey of country prevalence and practices. J Matern Fetal Neonatal Med.

[73] Uzan J, Carbonnel M, Piconne O, Asmar R, Ayoubi JM (2011). Pre-eclampsia: pathophysiology, diagnosis, and management. Vasc Health Risk Manag.

[74] Hutcheon JA, Lisonkova S, Joseph KS (2011). Epidemiology of pre-eclampsia and the other hypertensive disorders of pregnancy. Best Pract Res Clin Endocrinol Metab.

[75] Caughey AB, Stotland NE, Washington AE, Escobar GJ (2005). Maternal ethnicity, paternal ethnicity, and parental ethnic discordance: predictors of preeclampsia. Obstet Gynecol.

[76] Urquia ML, Ying I, Glazier RH, Berger H, De Souza LR, Ray JG (2012). Serious preeclampsia among different immigrant groups. J Obstet Gynaecol Can.

[77] Yogev, Chen, Hod, Coustan, Oats, McIntyre (2010). Hyperglycemia and Adverse Pregnancy Outcome (HAPO) study: preeclampsia. Am J Obstet Gynecol.

[78] Bouthoorn SH, Gaillard R, Steegers EA, Hofman A, Jaddoe VW, van Lenthe FJ (2012). Ethnic differences in blood pressure and hypertensive complications during pregnancy: the generation R study. Hypertension.

[79] Tanaka M, Jaamaa G, Kaiser M, Hills E, Soim A, Zhu M (2007). Racial disparity in hypertensive disorders of pregnancy in New York State: a 10-year longitudinal population-based study. Am J Public Health.

[80] Schmitt NM, Nicholson WK, Schmitt J (2007). The association of pregnancy and the development of obesity – results of a systematic review and meta-analysis on the natural history of postpartum weight retention. Int J Obes.

[81] Cheng HR, Walker LO, Tseng YF, Lin PC (2011). Post-partum weight retention in women in Asia: a systematic review. Obes Rev.

[82] van Poppel MN, Hartman MA, Hosper K, van Eijsden M (2012). Ethnic differences in weight retention after pregnancy: the ABCD study. Eur J Public Health.

[83] Blaudeau TE, Hunter GR, St-Onge M-P, Gower BA, Roy JLP, Bryan DR (2008). IAAT, catecholamines, and parity in African-American and European-American women. Obesity.

[84] Cohen SS, Larson CO, Matthews CE, Buchowski MS, Signorello LB, Hargreaves MK (2009). Parity and breastfeeding in relation to obesity among black and white women in the southern community cohort study. J Womens Health.

[85] Giroux I, Lander S, Charlesworth S, Mottola MF (2009). Weight history of overweight pregnant women. Can J Diet Pract Res.

[86] Davis EM, Zyzanski SJ, Olson CM, Stange KC, Horwitz RI (2009). Racial, ethnic, and socioeconomic differences in the incidence of obesity related to childbirth. Am J Public Health.

[87] Gunderson EP, Striegel-Moore R, Schreiber G, Hudes M, Biro F, Daniels S (2009). Longitudinal study of growth and adiposity in parous compared with nulligravid adolescents. Arch Pediatr Adolesc Med.

[88] Baker JL, Gamborg M, Heitmann BL, Lissner L, Sørensen TI, Rasmussen KM (2008). Breastfeeding reduces postpartum weight retention. Am J Clin Nutr.

[89] Krause KM, Lovelady CA, Peterson BL, Chowdhury N, Ostbye T (2010). Effect of breast-feeding on weight retention at 3 and 6 months postpartum: data from the North Carolina WIC Programme. Public Health Nutr.

[90] Onyango AW, Nommsen-Rivers L, Siyam A, Borghi E, de Onis M, Garza C (2011). Post-partum weight change patterns in the WHO Multicentre Growth Reference Study. Matern Child Nutr.

[91] Health and Social Care Information Centre, IFF Research (2012). Infant Feeding Survey UK 2010. Chapter 2, Incidence, prevalence and duration of breastfeeding. https://catalogue.ic.nhs.uk/publications/public-health/surveys/infant-feed-surv-2010/ifs-uk-2010-chap2-inc-prev-dur.pdf.

[92] van Rossem L, Vogel I, Steegers EA, Moll HA, Jaddoe VW, Hofman A (2010). Breastfeeding patterns among ethnic minorities: the Generation R Study. J Epidemiol Community Health.

[93] Hawkins SS, Lamb K, Cole TJ, Law C (2008). Influence of moving to the UK on maternal health behaviours: prospective cohort study. BMJ.

[94] WHO (2012). The WHO global data bank on infant and young child feeding. http://www.who.int/nutrition/databases/infantfeeding/en/index.html.

[95] Amir L, Donath S (2007). A systematic review of maternal obesity and breastfeeding intention, initiation and duration. BMC Pregnancy Childbirth.

[96] Wojcicki JM (2011). Maternal prepregnancy body mass index and initiation and duration of breastfeeding: a review of the literature. J Womens Health.

[97] Kim C, Berger DK, Chamany S (2007). Recurrence of gestational diabetes mellitus. Diabetes Care.

[98] Tovar A, Chasan-Taber L, Eggleston E, Oken E (2011). Postpartum screening for diabetes among women with a history of gestational diabetes mellitus. Prev Chronic Dis.

[99] Ratner RE, Christophi CA, Metzger BE, Dabelea D, Bennett PH, Pi-Sunyer X (2008). Prevention of Diabetes in women with a history of gestational diabetes: effects of metformin and lifestyle interventions. J Clin Endocrinol Metab.

[100] Kim C, Newton KM, Knopp RH (2002). Gestational diabetes and the incidence of type 2 diabetes. Diabetes Care.

[101] Retnakaran R, Austin PC, Shah BR (2011). Effect of subsequent pregnancies on the risk of developing diabetes following a first pregnancy complicated by gestational diabetes: a population-based study. Diabet Med.

[102] Vambergue A, Dognin C, Boulogne A, Réjou MC, Biausque S, Fontaine P (2008). Increasing incidence of abnormal glucose tolerance in women with prior abnormal glucose tolerance during pregnancy: DIAGEST 2 study. Diabet Med.

[103] Kakad R, Anwar A, Dyer P, Webber J, Dale J (2010). Fasting plasma glucose is not sufficient to detect ongoing glucose intolerance after pregnancy complicated by gestational diabetes. Exp Clin Endocrinol Diabetes.

[104] Bellamy L, Casas J-P, Hingorani AD, Williams D (2009). Type 2 diabetes mellitus after gestational diabetes: a systematic review and meta-analysis. Lancet.

[105] Feig DS, Zinman B, Wang X, Hux JE (2008). Risk of development of diabetes mellitus after diagnosis of gestational diabetes. CMAJ.

[106] Xiang AH, Li BH, Black MH, Sacks DA, Buchanan TA, Jacobsen SJ (2011). Racial and ethnic disparities in diabetes risk after gestational diabetes mellitus. Diabetologia.

[107] Girgis CM, Gunton JE, Cheung NW (2012). The influence of ethnicity on the development of type 2 diabetes mellitus in women with gestational diabetes: a prospective study and review of the literature. ISRN Endocrinol.

[108] Wang Y, Chen L, Horswell R, Xiao K, Besse J, Johnson J (2012). Racial differences in the association between gestational diabetes mellitus and risk of type 2 diabetes. J Women Health.

[109] Bo S, Valpreda S, Menato G, Bardelli C, Botto C, Gambino R (2007). Should we consider gestational diabetes a vascular risk factor?. Atherosclerosis.

[110] Carr DB, Utzschneider KM, Hull RL, Tong J, Wallace TM, Kodama K (2006). Gestational diabetes mellitus increases the risk of cardiovascular disease in women with a family history of type 2 diabetes. Diabetes Care.

[111] Shah BR, Retnakaran R, Booth GL (2008). Increased risk of cardiovascular disease in young women following gestational diabetes mellitus. Diabetes Care.

[112] Milne F, Redman C, Walker J, Baker P, Bradley J, Cooper C (2005). The pre-eclampsia community guideline (PRECOG): how to screen for and detect onset of pre-eclampsia in the community. BMJ.

[113] Savvidou MD, Hingorani AD, Tsikas D, Frolich JC, Vallance P, Nicolaides KH (2003). Endothelial dysfunction and raised plasma concentrations of asymmetric dimethylarginine in pregnant women who subsequently develop pre-eclampsia. Lancet.

[114] Chambers JC, Fusi L, Malik IS, Haskard DO, De Swiet M, Kooner JS (2001). Association of maternal endothelial dysfunction with preeclampsia. JAMA.

[115] Bellamy L, Casas JP, Hingorani AD, Williams DJ (2007). Pre-eclampsia and risk of cardiovascular disease and cancer in later life: systematic review and meta-analysis. BMJ.

[116] Hannaford P, Ferry S, Hirsch S (1997). Cardiovascular sequelae of toxaemia of pregnancy. Heart.

[117] Hanson MA, Low FM, Gluckman PD (2011). Epigenetic epidemiology: the rebirth of soft inheritance. Ann Nutr Metab.

[118] Hanson MA, Gluckman PD (2011). Developmental origins of health and disease: moving from biological concepts to interventions and policy. Int J Gynaecol Obstet.

[119] Lewis RM, Cleal JK, Hanson MA (2012). Review: placenta, evolution and lifelong health. Placenta.

[120] Dabelea D, Crume T (2011). Maternal environment and the transgenerational cycle of obesity and diabetes. Diabetes.

[121] Catalano PM (2003). Obesity and pregnancy – the propagation of a viscous cycle?. J Clin Endocrinol Metab.

[122] Goedhart G, van Eijsden M, van der Wal MF, Bonsel GJ (2008). Ethnic differences in term birthweight: the role of constitutional and environmental factors. Paediatr Perinat Epidemiol.

[123] Kelly Y, Panico L, Bartley M, Marmot M, Nazroo J, Sacker A (2009). Why does birthweight vary among ethnic groups in the UK? Findings from the Millennium Cohort Study. J Public Health.

[124] Moser K, Stanfield KM, Leon DA (2008). Birthweight and gestational age by ethnic group, England and Wales 2005: introducing new data on births. Health Stat Q.

[125] Harding S, Boroujerdi M, Santana P, Cruickshank J (2006). Decline in, and lack of difference between, average birth weights among African and Portuguese babies in Portugal. Int J Epidemiol.

[126] Harding S, Rosato MG, Cruickshank JK (2004). Lack of change in birthweights of infants by generational status among Indian, Pakistani, Bangladeshi, Black Caribbean, and Black African mothers in a British cohort study. Int J Epidemiol.

[127] Harding S, Santana P, Cruickshank JK, Boroujerdi M (2006). Birth weights of black African babies of migrant and nonmigrant mothers compared with those of babies of European mothers in Portugal. Ann Epidemiol.

[128] Drooger JC, Troe JWM, Borsboom GJJM, Hofman A, Mackenbach JP, Moll HA (2005). Ethnic differences in prenatal growth and the association with maternal and fetal characteristics. Ultrasound Obstet Gynecol.

[129] Vahratian A, Buekens P, Delvaux T, Boutsen M, Wang Y, Kupper LL (2004). Birthweight differences among infants of North African immigrants and Belgians in Belgium. Eur J Public Health.

[130] Vangen S, Stoltenberg C, Skjaerven R, Magnus P, Harris JR, Stray-Pedersen B (2002). The heavier the better? Birthweight and perinatal mortality in different ethnic groups. Int J Epidemiol.

[131] Troe E, Raat H, Jaddoe VWV, Hofman A, Looman CWN, Moll HA (2007). Explaining differences in birthweight between ethnic populations. The Generation R Study BJOG.

[132] Leary S, Fall C, Osmond C, Lovel H, Campbell D, Eriksson J (2006). Geographical variation in relationships between parental body size and offspring phenotype at birth. Acta Obstet Gynecol Scand.

[133] Modi N, Thomas EL, Uthaya SN, Umranikar S, Bell JD, Yajnik C (2009). Whole body magnetic resonance imaging of healthy newborn infants demonstrates increased central adiposity in Asian Indians. Pediatr Res.

[134] Yajnik CS, Fall CHD, Coyaji KJ, Hirve SS, Rao S, Barker DJP (2003). Neonatal anthropometry: the thin-fat Indian baby. The Pune Maternal Nutrition Study. Int J Obes Relat Metab Disord.

[135] Singh KA, Huston-Presley LP, Mencin P, Thomas A, Amini SB, Catalano PM (2010). Birth weight and body composition of neonates born to Caucasian compared with African-American mothers. Obstet Gynecol.

[136] Lampl M, Lee W, Koo W, Frongillo EA, Barker DJ, Romero R (2012). Ethnic differences in the accumulation of fat and lean mass in late gestation. Am J Hum Biol.

[137] Catalano PM, Thomas A, Huston-Presley L, Amini SB (2003). Increased fetal adiposity: a very sensitive marker of abnormal in utero development. Am J Obstet Gynecol.

[138] Catalano PM, Ehrenberg HM (2006). Review article: the short- and long-term implications of maternal obesity on the mother and her offspring. BJOG.

[139] Metzger BE, Lowe LP, Dyer AR, Trimble ER, Chaovarindr U, Coustan DR (2008). Hyperglycemia and adverse pregnancy outcomes. N Engl J Med.

[140] Catalano PM, McIntyre HD, Cruickshank JK, McCance DR, Dyer AR, Metzger BE (2012). The hyperglycemia and adverse pregnancy outcome study: associations of GDM and obesity with pregnancy outcomes. Diabetes Care.

[141] Hunt KJ, Marlow NM, Gebregziabher M, Ellerbe CN, Mauldin J, Mayorga ME (2012). Impact of maternal diabetes on birthweight is greater in non-Hispanic blacks than in non-Hispanic whites. Diabetologia.

[142] Makgoba M, Savvidou MD, Steer PJ (2012). The effect of maternal characteristics and gestational diabetes on birthweight. BJOG.

[143] Gruslin A, Lemyre B (2011). Pre-eclampsia: fetal assessment and neonatal outcomes. Best Pract Res Clin Endocrinol Metab.

[144] Catalano PM, Farrell K, Thomas A, Huston-Presley L, Mencin P, de Mouzon SH (2009). Perinatal risk factors for childhood obesity and metabolic dysregulation. Am J Clin Nutr.

[145] Brisbois TD, Farmer AP, McCargar LJ (2012). Early markers of adult obesity: a review. Obes Rev.

[146] Godfrey KM, Inskip HM, Hanson MA (2011). The long-term effects of prenatal development on growth and metabolism. Semin Reprod Med.

[147] Dewey KG, Peerson JM, Brown KH, Krebs NF, Michaelsen KF, Persson LA (1995). Growth of breast-fed infants deviates from current reference data: a pooled analysis of US, Canadian, and European data sets. World Health Organization Working Group on Infant Growth Pediatrics.

[148] de Hoog ML, van Eijsden M, Stronks K, Gemke RJ, Vrijkotte TG (2011). The role of infant feeding practices in the explanation for ethnic differences in infant growth: the Amsterdam Born Children and their Development study. Br J Nutr.

[149] de Hoog ML, van Eijsden M, Stronks K, Gemke RJ, Vrijkotte TG (2011). Overweight at age two years in a multi-ethnic cohort (ABCD study): the role of prenatal factors, birth outcomes and postnatal factors. BMC Public Health.

[150] Kramer MS, Matush L, Vanilovich I, Platt R, Bogdanovich N, Sevkovskaya Z (2007). Effect of prolonged and exclusive breast feeding on risk of allergy and asthma: cluster randomised trial. BMJ.

[151] Brion MJA, Lawlor DA, Matijasevich A, Horta B, Anselmi L, Araújo CL (2011). What are the causal effects of breastfeeding on IQ, obesity and blood pressure? Evidence from comparing high-income with middle-income cohorts. Int J Epidemiol.

[152] Beyerlein A, von Kries R (2011). Breastfeeding and body composition in children: will there ever be conclusive empirical evidence for a protective effect against overweight?. Am J Clin Nutr.

[153] Crume TL, Bahr TM, Mayer-Davis EJ, Hamman RF, Scherzinger AL, Stamm E (2012). Selective protection against extremes in childhood body size, abdominal fat deposition, and fat patterning in breastfed children. Arch Pediatr Adolesc Med.

[154] Fall CH, Borja JB, Osmond C, Richter L, Bhargava SK, Martorell R (2011). Infant-feeding patterns and cardiovascular risk factors in young adulthood: data from five cohorts in low- and middle-income countries. Int J Epidemiol.

[155] Gillman MW (2011). Commentary: breastfeeding and obesity – the 2011 scorecard. Int J Epidemiol.

[156] Arenz S, Ruckerl R, Koletzko B, von Kries R (2004). Breast-feeding and childhood obesity – a systematic review. Int J Obes Relat Metab Disord.

[157] Owen CG, Martin RM, Whincup PH, Davey-Smith G, Gillman MW, Cook DG (2005). The effect of breastfeeding on mean body mass index throughout life: a quantitative review of published and unpublished observational evidence. Am J Clin Nutr.

[158] Harder T, Bergmann R, Kallischnigg G, Plagemann A (2005). Duration of breastfeeding and risk of overweight: a meta-analysis. Am J Epidemiol.

[159] Owen CG, Martin RM, Whincup PH, Smith GD, Cook DG (2005). Effect of infant feeding on the risk of obesity across the life course: a quantitative review of published evidence. Pediatrics.

[160] Savino F, Liguori S, Fissore M, Oggero R (2009). Breast milk hormones and their protective effect on obesity. Int J Pediatr Endocrinol.

[161] Escribano J, Luque V, Ferre N, Mendez-Riera G, Koletzko B, Grote V (2012). Effect of protein intake and weight gain velocity on body fat mass at 6 months of age: the EU childhood obesity programme. Int J Obes.

[162] Li R, Fein SB, Grummer-Strawn LM (2010). Do infants fed from bottles lack self-regulation of milk intake compared with directly breastfed infants?. Pediatrics.

[163] Hales CN, Barker DJ, Clark PM, Cox LJ, Fall C, Osmond C (1991). Fetal and infant growth and impaired glucose tolerance at age 64. BMJ.

[164] Whincup PH, Kaye SJ, Owen CG, Huxley R, Cook DG, Anazawa S (2008). Birth weight and risk of type 2 diabetes. JAMA.

[165] Owen CG, Martin RM, Whincup PH, Smith GD, Cook DG (2006). Does breastfeeding influence risk of type 2 diabetes in later life? A quantitative analysis of published evidence. Am J Clin Nutr.

[166] D'Adamo E, Caprio S (2011). Type 2 diabetes in youth: epidemiology and pathophysiology. Diabetes Care.

[167] Dabelea D, Bell RA, D'Agostino RB, Imperatore G, Johansen JM, Linder B (2007). Incidence of diabetes in youth in the United States. JAMA.

[168] El-Sayed AM, Scarborough P, Galea S (2011). Ethnic inequalities in obesity among children and adults in the UK: a systematic review of the literature. Obes Rev.

[169] Nightingale CM, Rudnicka AR, Owen CG, Cook DG, Whincup PH (2011). Patterns of body size and adiposity among UK children of South Asian, black African-Caribbean and white European origin: Child Heart And health Study in England (CHASE Study). Int J Epidemiol.

[170] Whincup PH, Nightingale CM, Owen CG, Rudnicka AR, Gibb I, McKay CM (2010). Early emergence of ethnic differences in type 2 diabetes precursors in the UK: the Child Heart and Health Study in England (CHASE Study). PLoS Med.

[171] Weiss R, Caprio S, Trombetta M, Taksali SE, Tamborlane WV, Bonadonna R (2005). Β-cell function across the spectrum of glucose tolerance in obese youth. Diabetes.

[172] Risnes KR, Vatten LJ, Baker JL, Jameson K, Sovio U, Kajantie E (2011). Birthweight and mortality in adulthood: a systematic review and meta-analysis. Int J Epidemiol.

[173] Tran A, Straand J, Diep L, Meyer H, Birkeland K, Jenum A (2011). Cardiovascular disease by diabetes status in five ethnic minority groups compared to ethnic Norwegians. BMC Public Health.

[174] Yusuf S, Reddy S, Ounpuu S, Anand S (2001). Global burden of cardiovascular diseases: part II: variations in cardiovascular disease by specific ethnic groups and geographic regions and prevention strategies. Circulation.

[175] Lynch J, Smith GD (2005). A life course approach to chronic disease epidemiology. Ann Rev Public Health.

[176] World Health Organization (2002). Antenatal care randomized trial: manual for the implementation of the new model.

[177] Dodd JM, Grivell RM, Crowther CA, Robinson JS (2010). Antenatal interventions for overweight or obese pregnant women: a systematic review of randomised trials. BJOG.

[178] Renzaho AMN, Skouteris H, Oldroyd J (2010). Preventing gestational diabetes mellitus among migrant women and reducing obesity and type 2 diabetes in their offspring: a call for culturally competent lifestyle interventions in pregnancy. J Am Diet Assoc.

[179] Ronnberg AK, Nilsson K (2010). Interventions during pregnancy to reduce excessive gestational weight gain: a systematic review assessing current clinical evidence using the Grading of Recommendations, Assessment, Development and Evaluation (GRADE) system. BJOG.

[180] Kuhlmann AKS, Dietz PM, Galavotti C, England LJ (2008). Weight-management interventions for pregnant or postpartum women. Am J Prev Med.

[181] Streuling I, Beyerlein A, von Kries R (2010). Can gestational weight gain be modified by increasing physical activity and diet counseling?. A meta-analysis of interventional trials Am J Clin Nutr.

[182] Gardner B, Wardle J, Poston L, Croker H (2011). Changing diet and physical activity to reduce gestational weight gain: a meta-analysis. Obes Rev.

